# A trial studying approach to predict college achievement

**DOI:** 10.3389/fpsyg.2015.00887

**Published:** 2015-06-30

**Authors:** Rob R. Meijer, A. Susan M. Niessen

**Affiliations:** Psychometrics and Statistics, Faculty of Behavioral and Social Sciences, University of GroningenGroningen, Netherlands

**Keywords:** selection in higher education, work sample, achievement testing, college achievement, selection

## Abstract

We argue that using trial studying is a reliable and valid way to select students for higher education. This method is based on a work sample approach often used in personnel selection contexts. We discuss that this method has predictive validity for study success, has high acceptance by stakeholders, and measures self-regulation in a high-stakes testing context that cannot be measured through self-report questionnaires. We suggest further research to implement this method to select students.

Standardized tests are excellent tools to predict study success in college and graduate school (Kuncel and Hezlett, 2007). However, in modern society college entrance and admission tests should not only predict college achievement, but should also consists of tasks that reflect what has been learned in high school and tasks that are required in college: tests should measure “college preparedness” (The College Board, 2014). For example, in the USA the SAT has been criticized because it contained too many questions related to some form of intelligence testing and questions unrelated to the high school curriculum (Lemann, 1999). Furthermore, as discussed in Balf (2014) high school students should not be focused on test preparation, but on learning and acquiring skills. In the new SAT, the aim is therefore to enhance the relevance of the questions for success in college, whereas at the same time keeping the predictive validity intact (The College Board, 2014).

In several European countries college entrance testing has become more important due to increasing numbers of students participating in higher education (Osborn, 2003). For these high-stakes tests the acceptance of stakeholders is of great importance and, thus, besides predictive validity, content validity, and face validity are important criteria for a good test^1^. Now that selective college admission in Europe is becoming more popular, we argue that a “work sample,” in the form of trial studying, is a good method to select students for higher education because it has high predictive, content, and face validity. Before discussing this approach we first discuss shortly other popular methods to select students.

## Predictors for academic achievement

As an alternative to standardized tests like the SAT or ACT, high school grades have good predictive validity for academic achievement when applicants have similar educational backgrounds and they meet the criteria of face validity and content validity (Atkinson and Geiser, 2009). However, in countries that do not have a national curriculum, “high schools differ widely in their grading standards and grades reflect accomplishment within a high school, but are not comparable across high schools” (Trautwein and Baeriswyl, 2007; Zwick, 2013). Furthermore, even in countries with a national curriculum, such as most Western European countries, high-school grades are often difficult to compare across college applicants. Applicants followed different levels of high school programs, come from different countries with different grading systems, or start higher education at a later age so that grades reflect different knowledge levels and do not include knowledge obtained in, for example, jobs. In Germany, for example, a national selection system would be unfair when student selection will be conducted on the basis of grades because different states within the country use grading systems that differ in difficulty level.

For admission to higher professional education like medicine and law, students are administered tests that are more related to the study of interest. For example, the Law School Admission Test consists of subtests of different types of verbal reasoning and reading tasks that are strongly related to the lengthy and complex-reasoning skills commonly encountered in law school. Kuncel and Hezlett (2007) reviewed several studies and meta-analyses in predicting graduate school success and concluded that the strongest predictors were tests that were specifically linked to the discipline of interest. However, they also discussed that “Student motivation and interest, which are critical for sustained effort though graduate education, must be inferred from various unstandardized measures including letters of recommendation, personal statements, and interviews. Additional research is needed to develop measures that provide more reliable information about these key characteristics.”

Thus, we need instruments that measure both achievement and sustained effort and most important: we need measures that are not easy to fake. Existing self-report questionnaires are not very useful in a high-stakes context given the susceptibility of such questionnaires to faking and socially desirable responding.

## Trial studying approach

In some recent studies, discussed below, measurement of “college preparedness” and “student self-regulation” (Cohen, 2012) was combined. Self-regulation refers to the self-generated thoughts, feelings, and actions for attaining one's goal^2^. In these studies tests were used that were closely linked to a particular study.

Niessen et al. (submitted) investigated a strategy to select students inspired by Visser et al. (2012). Visser et al. (2012) studied a method they called “trial studying” for selecting students for a psychology program, which was based on the observation that the best predictor for performance in undergraduate higher education was the first grade obtained in higher education^1^. Therefore, they developed an admission test that was similar to the first course in the psychology program. This admission test consisted of attending a lecture, studying material from an introductory psychology course, and, taking an exam on the content of both the lecture and the material. Because students had to study material from an introductory book and had to attend the test day, this measures both cognitive ability as well as self-regulation. This approach is similar to the well-known “work sample” approach (Visser et al., 2012) in personnel selection were a sample of future expected behavior (e.g., leaderless group decision tasks) is taken as a predictor for future success on the job (being a good leader). It also has similarities with a portfolio approach. However, only in the case when the portfolio represents assignments that are similar as the assignments that are given in the study, so for example, in art studies. Visser et al. (2012) found that students who were admitted to the program after passing this test performed better than students who were admitted without passing the test.

Niessen et al. (submitted) used a test that mimicked the first course in a psychology program, so that the test had a high similarity to tasks that students are expected to perform. This test showed a high predictive validity (*r* = 0.49) and significant incremental validity over high-school grades in predicting first year mean grade (Δ*R^2^* = 0.04). There are a number of advantages to this method. First, predictive validity is high because the predictor and the criterion measures are similar. Second, the acceptance of stakeholders is expected to be much higher than for other trait-based measures like intelligence and personality. Showing a future student that a representative exam is too difficult (regardless of the underlying reason, whether it was ability or study-strategies or something else) and arguing that this is what is expected from students, is much more convincing than saying, for example, that his/her verbal reasoning score is not optimal for the study of interest. This is not to say as one reviewer correctly emphasized that “if a student does not succeed initially, they will likely never succeed.” Through better study strategies or, for example, help from experienced peers it is possible to improve scores. In fact, the trial studying approach emphasizes shown behavior instead of “innate abilities.”

Third, providing students with discipline-specific material that they should study may have an important self-regulated component. Although self-regulation can be measured using, for example, standardized self-report measures, this is often difficult in a high-stakes context because these questionnaires are easy to fake. Using behavioral measures is therefore a more convincing indicator of effective self-regulation than a self-report measure. Studying material and investing time and effort to do well are essential for future success. Using a work sample approach allows selecting students based on shown behavior, represented by shown effort to study the material. This approach follows the message of stakeholders like students, educators, admission officers, and parents that as Lemann (Kuncel and Hezlett, 2007) described it “life is about how hard you work and how much you learn.” Indeed, Schripsema et al. (2014) found that applicants for medical school who chose to take part in an extensive selection procedure had higher first year GPA scores than students who chose to be admitted through a lottery, probably due to higher motivation or self-regulation.

Finally, like the use of high school GPA, the use of a work sample approach may help to increase ethnic diversity. In an overview of personnel selection research Callinan and Robertson (2000) discussed that a work sample approach reduced adverse impact against minorities groups compared to traditional cognitive measures.

There are three common criticisms of the work-sample approach. The first is that college admission tests should not only be about predicting academic achievement. Social skills, integrity, and leadership, for example, are also considered to be valuable traits. Standardized tests like the SAT, but also the work sample test described above do not take these skills into account. However, for disciplines where such skills are of great importance, a work sample approach can be used to test these skills. An example is the use of multiple mini-interview (MMI) in admission to medical school (Eva et al., 2004). The MMI consists of a series of short structured clinical interviews and tasks where test takers show their interpersonal skills and ethical standards. These tasks can also be perceived as work samples for the courses on patient- or colleague interactions and on the use of these skills after graduation, as practitioners. Thus, using a work sample approach in higher education does not have to be limited to sampling “classic” student behaviour like studying books.

Another challenge is what exactly is being measured in a work sample. Some argue that since we do not know exactly what traits are being measured, and to what extent they influence test scores, the test lacks construct validity. We argue, however, that when you can reliably sample the criterion behaviour, we do not need traits. In a modern society we need tests with relevant content that are acceptable and explainable to the stakeholders.

Finally, we think that the work sample approach is especially suitable when selecting student for specific programs. In the European university context for instance, students are selected to follow a specific program such as psychology, law or medicine at the bachelor level, in contrast to the US system where students are selected for college in general. In addition, European students are often already pre-selected on learning abilities due to a high level of educational stratification, making tests that measure general skills less suitable. However, we do think that also for selecting students for more general undergraduate programs in the USA, the method may be interesting to consider. For example, universities may use this method in combination with college entrance test scores like the SAT or ACT to select students with special talents, underrepresented ethnic groups, or to select the most motivated students.

### Conflict of interest statement

The authors declare that the research was conducted in the absence of any commercial or financial relationships that could be construed as a potential conflict of interest.

## Footnotes

^1^In Western Europe students apply directly for a specific discipline. Students often choose a specific program in which they major from the beginning of undergraduate education and they often apply directly to the educational program (e.g., medicine, psychology, law). This is comparable to admission practices for professional and graduate education in, for example, the USA.

^2^In a first draft of this study we used the term motivation as a general term that simply refers to “how eager an applicant is to start with the study in a particular field.” As one of the reviewers noted motivation is context depended and multifaceted. We do not deny this, and it is perfectly possible that a student becomes less motivated during the study, or that a student is motivated for different reasons (to learn new things or to get rich). Therefore, the construct of self-regulation provides a better description of what the trial study is measuring.
